# Procalcitonin-guided algorithm to reduce length of antibiotic therapy in patients with severe sepsis and septic shock

**DOI:** 10.1186/1471-2334-13-158

**Published:** 2013-04-01

**Authors:** Andreas Hohn, Stefan Schroeder, Anna Gehrt, Kathrin Bernhardt, Berthold Bein, Karl Wegscheider, Marcel Hochreiter

**Affiliations:** 1 Department of Anaesthesiology, Intensive Care, Palliative Care and Pain Medicine, BG University Hospital Bergmannsheil, Ruhr-University Bochum, Bürkle-de-la-Camp-Platz 1, Bochum 44789, Germany; 2 Department of Anaesthesiology, Intensive Care Medicine and Pain Therapy, Hospital Düren gem. GmbH, Roonstr. 30, Düren 52351, Germany; 3 Department of Anaesthesiology and Intensive Care Medicine, West Coast Hospital, Esmarchstr. 50, Heide 25746, Germany; 4 Department of Anaesthesiology and Intensive Care Medicine, University Hospital Schleswig-Holstein, Campus Kiel, Schwanenweg 21, Kiel 24105, Germany; 5 Department of Medical Biometry and Epidemiology, University Medical Center Hamburg-Eppendorf, Martinistr. 52, Hamburg 20246, Germany; 6 Department of Anaesthesiology, University Hospital Heidelberg, Im Neuenheimer Feld 110, Heidelberg 69120, Germany

**Keywords:** Procalcitonin, Sepsis, Economical outcomes, Intensive care

## Abstract

**Background:**

Procalcitonin (PCT)-protocols to guide antibiotic treatment in severe infections are known to be effective. But less is known about the long-term effects of such protocols on antibiotic consumption under real life conditions. This retrospective study analyses the effects on antibiotic use in patients with severe sepsis and septic shock after implementation of a PCT-protocol.

**Methods:**

We conducted a retrospective ICU-database search for adult patients between 2005 and 2009 with sepsis and organ dysfunction who where treated accordingly to a PCT-guided algorithm as follows: Daily measurements of PCT (BRAHMS PCT LIA®; BRAHMS Aktiengesellschaft, Hennigsdorf, Germany). Antibiotic therapy was discontinued if 1) clinical signs and symptoms of infection improved and PCT decreased to ≤1 ng/ml, or 2) if the PCT value was >1 ng/ml, but had dropped to 25-35% of the initial value within three days. The primary outcome parameters were: antibiotic days on ICU, ICU re-infection rate, 28-day mortality rate, length of stay (LOS) in ICU, mean antibiotic costs (per patient) and ventilation hours. Data from 141 patients were included in our study. Primary outcome parameters were analysed using covariance analyses (ANCOVA) to control for effects by gender, age, SAPS II, APACHE II and effective cost weight.

**Results:**

From baseline data of 2005, duration of antibiotic therapy was reduced by an average of 1.0 day per year from 14.3 ±1.2 to 9.0 ±1.7 days in 2009 (p=0.02). ICU re-infection rate was decreased by yearly 35.1% (95% CI −53 to −8.5; p=0.014) just as ventilation hours by 42 hours per year (95% CI −72.6 to −11.4; p=0.008). ICU-LOS was reduced by 2.7 days per year (p<0.001). Trends towards an average yearly reduction of 28-day mortality by −22.4% (95% CI −44.3 to 8.1; p=0.133) and mean cost for antibiotic therapy/ patient by −14.3 Euro (95% CI −55.7 to 27.1) did not reach statistical significance.

**Conclusions:**

In a real-life clinical setting, implementation of a PCT-protocol was associated with a reduced duration of antibiotic therapy in septic ICU patients without compromising clinical or economical outcomes.

**German clinical trials register:**

DRKS00003490

## Background

There is a dramatic increase in antibiotic-resistant infections [1-3], leading to higher mortality, longer hospital or intensive care unit (ICU) stays, and increased hospital costs [3,4]. While timely initiation of antibiotic therapy in patients with severe sepsis and septic shock is crucial and has been proven [5], length of antibiotic treatment in clinical practice often is decided by the attending clinician by means of his experience.

But, shortening the length of antibiotic courses seems to play an important role in reducing antibiotic resistance [6] and its effectiveness has been shown in several studies [7-9]. In this context, there is rising evidence that a procalcitonin (PCT)- based algorithm is a useful tool to support clinical decision when tailoring antibiotic therapy. Several randomised controlled trials in a variety of settings showed a reduction in antibiotic duration or an increase in antibiotic-free days. Clinical outcome either remained unaffected [10-15] or even was improved, as two studies revealed a reduction of length of stay (LOS) in the ICU [16,17]. Furthermore, recent meta-analyses concluded that PCT-guided antibiotic treatment appears to be safe, reduces antibiotic consumption and may also improve outcome [18-20]. In patients with acute respiratory infections, a current systematic Cochrane review revealed a significant reduction in duration of antibiotic treatment without negative effects on clinical outcome parameters [21].

Although the effectiveness of PCT-guided antibiotic therapy has been proven in several randomised clinical trials, so far less is known about implementation and effects of such protocols under out-of-study conditions. For patients with respiratory infections outside the ICU, two studies demonstrated that following a PCT algorithm significantly reduces antibiotic use without increasing the risk of complications [22,23]. But so far there is no study that evaluates the long-term effects of such an algorithm on antibiotic consumption in surgical ICU patients. Thus, in the present study we analyse the development of duration of antibiotic treatment in patients with severe sepsis in a surgical intensive care unit (ICU) over a five years period after implementation of a PCT-guided algorithm.

## Methods

### Patients and data collection

Ethics commission approval was obtained from the Medical Faculty at Christian Albrecht University of Kiel (D409/10) for our trial in the surgical intensive care ward at the West Coast Hospital in Heide. The informed consent was waived in view of the retrospective and anonymous nature of the study.

**Figure 1:**
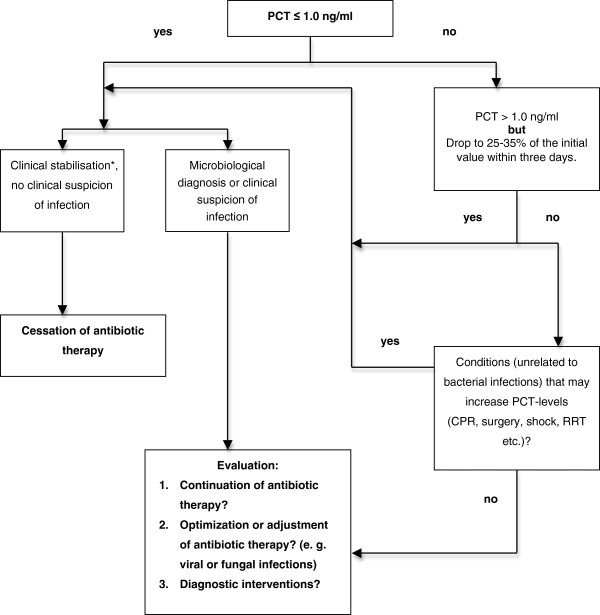
**PCT-algorithm.** PCT-algorithm used in clinical practice during the study period. PCT, procalcitonin. CPR, cardiopulmonary resuscitation. RRT, renal replacement therapy. * Clinical stabilisation: Haemodynamic stability, improvement of respiratory and renal function,stable metabolic state, improvement of lactat acidosis, stabilisation of mental state etc.

In 2005 we introduced a PCT-guided algorithm as follows: Daily measurements of PCT (BRAHMS PCT LIA®; BRAHMS Aktiengesellschaft, Hennigsdorf, Germany) with the daily routine blood samples. Antibiotic therapy was discontinued if 1) clinical signs and symptoms of infection improved and PCT decreased to ≤1 ng/ml, or 2) if the PCT value was >1 ng/ml, but had dropped to 25-35% of the initial value within three days (Figure 1).

We conducted a retrospective ICU-database search for adult patients from our surgical ICU between 2005 and 2009 with a) sepsis and b) organ dysfunction on presentation as defined by the ACCP/SCCM Consensus Conference Committee [24] (i.e. gas exchange impairment (partial pressure of arterial oxygen (PaO_2_)/fraction of inspired oxygen (FiO_2_) < 300 mmHg), acute renal dysfunction (2-fold baseline creatinine increase or urine output < 0.5 ml/kg for at least two hours), platelet count below 100,000 cells/mm^3^, lactate blood concentration above 2 mmol/l or sepsis induced impaired mental status. Patients with sepsis induced mean arterial pressure below 65 mmHg for ≥ 1 hour despite adequate fluid resuscitation were classified as having septic shock).

Exclusion criteria were: age under 18 years; known pregnancy; bone-marrow transplant or patient undergoing chemotherapy; infections for which long-term antibiotic treatment is strongly recommended (e.g. infective endocarditis, tuberculosis, anterior mediastinitis after cardiac surgery) and do-not-resuscitate orders. Also, patients with an incomplete data set were excluded.

Data were collected for gender, age, Simplified Acute Physiology Score (SAPS) II, Acute Physiology And Chronic Health Evaluation (APACHE) Score II, German Diagnosis Related Groups (G-DRG)-effective cost weight and for the primary outcome parameters: antibiotic days on ICU, ICU re-infection rate, LOS in ICU, ventilation hours, 28-day mortality, and antibiotic costs per patient. Re-infection was defined as the growth of the initial causative bacterial strains, taken from the same infection site after ≥ 48 h of stopping antibiotics, in addition to clinical signs or symptoms of infection. Mean antibiotic costs per patient (Euro) were calculated by means of the hospital’s wholesale prices without costs of administration.

### Data analysis

Descriptive statistics are mean ± standard deviation for patient characteristics or mean ± standard error or 95% confidence intervals (CI) for outcome. Chi-squared test was employed for comparison of infection sites over the years. Number of PCT-measurements per patient for trend significance over the years was analysed with Kruskal-Wallis test. Annual means/ percentages of patient characteristics were first compared for any difference by analysis of variance (ANOVA) for continuous characteristics and by logistic regression for categorical characteristics. P values of the corresponding F tests and Wald chi-square tests are reported. In a second step, polynomial contrasts and Cochran-Armitage tests were applied to test whether linear trends were to be observed in patient characteristics over the total study period. Since in observational studies of moderate size even non-significant imbalances may bias the results to a relevant extent, outcome analysis was exclusively performed by statistical control of the baseline characteristics. Thus, outcome parameters were tested for linear trends by analysis of covariance (ANCOVA) or logistic regression models with adjustment for the covariates gender, age, SAPS II, APACHE II and effective G-DRG cost weight. A p < 0.05 was considered statistically significant.

This method proves to be useful when a retrospective comparison between different treatment groups is performed as it compensates for imbalances in important prognostic variables, which may lead to biases in treatment effect, and additionally reduces the residual variability in the statistical models, thus increasing the precision of the estimates and the power of the applied statistical tests.

The statistical software package SPSS 15.0 (SPSS Inc., Chicago, IL, USA) and STATA 11.0 (STATACorp LP, College Station, TX, USA) were used for statistical analysis.

## Results

**Figure 2:**
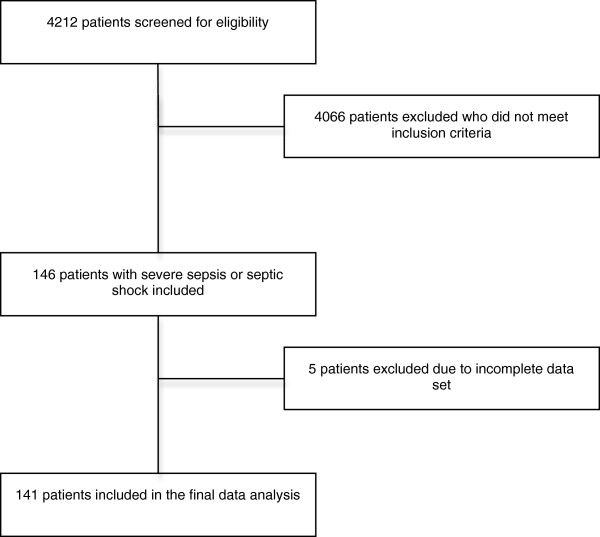
Screening and inclusion process.

Data of 4212 ICU-patients from 2005 to 2009 were screened. Through our data filter, we identified 146 patients with severe sepsis or septic shock. Of these, 5 patients were excluded from the study because of incomplete data set. In total, 141 patients were included in the final analysis set (Figure 2). Comparing the five years of study period, the number of patients, gender, age, SAPS II, APACHE II and effective cost weight fluctuated. However, no significant differences were observed (p > 0.05) with the exception of a linear trend in APACHE II towards lower means (p = 0.041) (Table 1). Over the years there was no significant difference between the infection sites (Table 2). For number of PCT measurements per patient, there was significant increasing trend from 2005 to 2009.

**Table 1:** Demographic, clinical data and primary outcome parameters

**Year**	**2005**	**2006**	**2007**	**2008**	**2009**	**P**^**§**^	**p-value trend**^**#**^	
**Patient** (n)	38	31	27	27	18			
**Gender** (m/f)	20/18	16/15	14/13	18/9	11/7	0.728	0.289	
**Age** (years)*	68.6 ±15.9	73.8 ±9.5	68.0 ±13.5	67.5 ±13.9	65.4 ±11.6	0.196	0.125	
SAPS II *	45.0 ±17.7	43.4 ±17.9	42.5 ±19.8	44.3 ±19.0	37.0 ±10.9	0.603	0.177	
Apache II *	20.0 ±8.8	20.5 ±8.8	18.2 ±8.7	17.9 ±9.7	15.6 ±7.2	0.327	0.041	
Effective cost weight *	14.8 ±13.6	13.3 ±11.4	17.2 ±14.0	14.5 ±13.6	19.2±18.5	0.623	0.256	
						**Average change per year**		
						**Mean**	**95% CI**	**p**
**ICU stay** (days)	26.0 ± 1.9	19.5 ± 2.2	18.5 ± 2.3	16.3 ± 2.3	15.8 ± 2.8	−2.7	−4.1 to −1.3	< 0.001
**Ventilation** (hours)	571.4 ± 41.5	455.0 ± 47.3	404.1 ± 49.2	362.0 ± 49.9	467.9 ± 60.9	−42.0	−72.6 to −11.4	0.008
**Antibiotic use** (days)	14.3 ± 1.2	12.0 ± 1.3	13.8 ± 1.4	12.1 ± 1.4	9.0 ± 1.7	−1.0	−1.9 to −0.2	0.02
**Antibiotic costs** (EUR/patient)	407.6 ± 57.2	420.7 ± 66.7	426.6 ± 67.7	393.2 ± 68.5	373.9 ± 83.7	−14.3	−55.7 to 27.1	0.495
**ICU reinfection rate** (proportion)	0.31 ± 0.07	0.37 ± 0.08	0.13 ± 0.06	0.12 ± 0.06	0.08 ± 0.06	−35.1%	−53.9% to −8.5%	0.014
**28-day mortality** (proportion)	0.54 ± 0.06	0.51 ± 0.07	0.55 ± 0.08	0.48 ± 0.07	0.34 ± 0.01	−22.4%	−44.3% to 8.1%	0.133

*Demographic and clinical data*:

* Mean ± standard deviation.

^§^ F test of ANOVA (continuous characteristic) or Wald chi square test (categorical characteristic) of logistic regression model.

^#^ Linear polynomial contrasts (continuous characteristic) or Cochran-Armitage trend tests (categorical characteristic).

SAPS II, Simplified Acute Physiology Score II.

APACHE II, Acute Physiology And Chronic Health Evaluation Score II.

*Primary outcome parameters*:

Adjusted Means ± standard error and average yearly change of outcome parameters, with 95 confidence limits (CI) and p values of trend tests from ANCOVA or logistic regression models with covariates gender, age, SAPS II, APACHE II and effective cost weight.

**Table 2:** Sites of infection

	**2005**	**2006**	**2007**	**2008**	**2009**	**p-value**
**Number of patients**	38	31	27	27	18	
**Pneumonia**	21,1% (8)	12,9% (4)	29,6% (8)	22,2% (6)	33,3% (6)	n. s.
**Abdominal**	57,9% (22)	58,1% (18)	63,0% (17)	53,6% (15)	38,9% (7)	n. s.
**Urogenital**	2,6% (1)	3,2% (1)	3,7% (1)	3,7% (1)	-	n. s.
**Soft tissue**	-	6,5% (2)	-	-	11,1% (2)	n. s.
**Others/ unknown**	18,4% (7)	19,4% (6)	3,7% (1)	18,5% (5)	16,7% (3)	n. s.

n. s., not significant.

**Figure 3:**
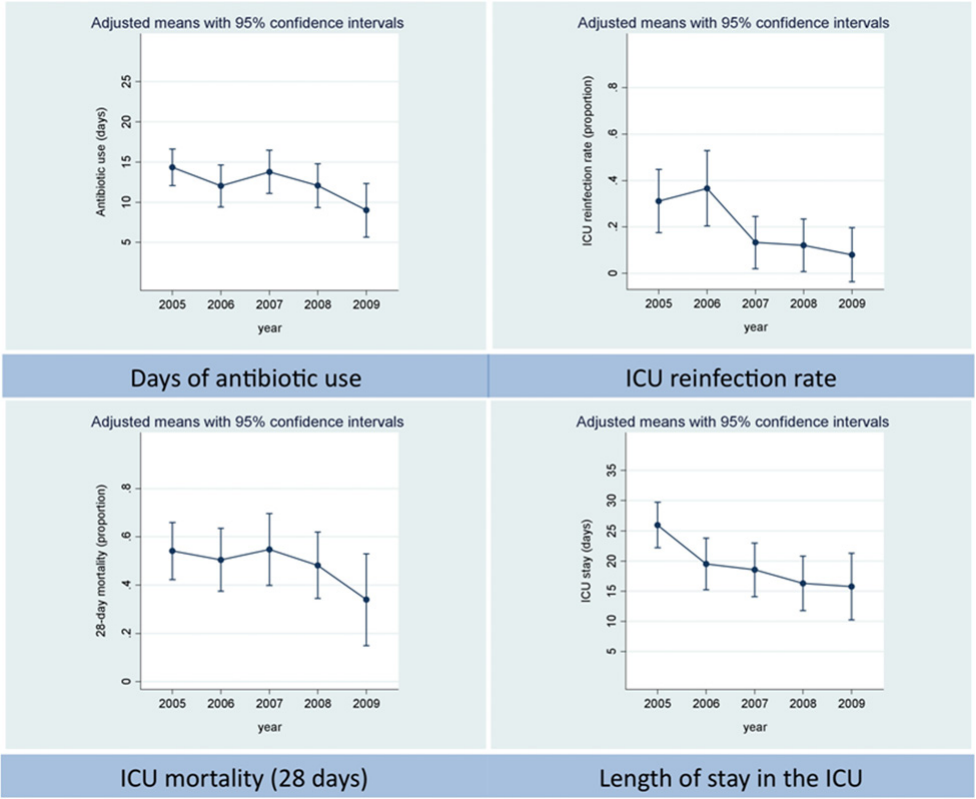
**Primary outcome parameters, graphical presentation.** Annual number of days of antibiotic use, ICU re-infection rate, 28-day mortality and length of ICU stay: means with 95% confidence intervals, adjusted for gender, age, SAPS II, APACHE II and effective cost weight.

The adjusted analysis of 6 outcome parameters from baseline of 2005 revealed significant average yearly reductions in days on antibiotic therapy (1.0 day per year, p = 0.02) from 14.3 ±1.2 to 9.0 ±1.7 days, rate of re-infection on ICU (35.1% per year, p = 0.014), ICU-LOS (2.7 days per year, p < 0.001) and ventilation time (42 hours per year, p = 0.008). Trends towards reduction of 28-day mortality by 22.4% per year (95% confidence interval: -44.3% to 8.1%; p = 0.133) and total cost for antibiotic therapy (14.3 Euro per patient and year, p = 0.495) per patient did not reach statistical significance (Table 1, Figure 3). Of the controlled covariates, effective cost weight had significant impact on all primary outcome parameters (p < 0.001). Effective cost weight represents the amount of reimbursement for the respective payment in the German Diagnosis Related Groups (G-DRG) system. It reflects resource consumption and clinical complexity of the hospital patient population. Furthermore, case mix index (i. e. the total cost weights of all inpatients per a defined time period divided by the number of admissions) is a surrogate marker for average patients’ clinical complexity and is correlated with antibiotic use in hospitals [25]. Age had significant impact on 28-day mortality (p < 0.001) whereas, for gender, SAPS II and APACHE II we found no significant impact on the primary outcome parameters.

## Discussion

### Antibiotic treatment

The results of the present study show that after implementation of a PCT-guided algorithm in 2005 length of antibiotic therapy in surgical patients with severe sepsis or septic shock was reduced by an average of 1.0 day per year from 14.3 ± 1.2 (2005) to 9.0 ± 1.7 days (2009). This reduction was associated with a significant reduction in ICU re-infection rate, ICU-LOS and ventilation hours and non-significant reduction of 28-day mortality and mean costs of antibiotics per patient. Thus, shortening antibiotic therapy did not have negative effects on clinical or economical outcome.

The length of antibiotic therapy was effectively reduced in several randomised controlled trials using PCT-guided algorithms. Thus, it was concluded that implementation of a PCT-based algorithm may reduce antibiotic exposure in critically ill, septic patients without compromising clinical outcomes [19]. Hence, our findings are in accordance with results from randomised controlled trials and further expand previous studies which confirmed the effectiveness of PCT-guided antibiotic treatment for non-ICU patients outside study conditions [22,23].

### Algorithm adherence

However, little is known about transfer of such protocols into clinical practice and development of antibiotic use after implementation of a PCT-guided algorithm. Implementation of new protocols in an ICU is complex, multi-professional, and transfer to clinical practice and optimisation of adherence is time-consuming and develops over a long period. For example, variability in compliance with sepsis resuscitation bundles has recently been shown [26]. And in a large one-day cross-sectional survey in German ICUs a great discrepancy between perception of guideline-adherence and clinical practice was found [27]. Two studies outside the ICU revealed a high adherence to the PCT algorithm and confirmed results from RCTs with a shortened duration of antibiotic treatment [22,23]. But in one study, a decreased compliance to the algorithm with increasing severity of illness was found [22]. This is consistent with RCTs in ICU patients where physicians refused to stop antibiotics despite the opposite suggestion of the PCT-algorithm in 16-53% [10,15,17]. In our analysis, adherence to the PCT-algorithm was not evaluated over the study period. But increasing average number of PCT-measurements per patient from 1.6 ±4.5 in 2005 to 18.6 ±16.4 in 2009, possibly indicates more acceptance of the PCT-protocol. Further studies are required to evaluate protocol-adherence in critically ill patients outside study conditions.

### Re-infection rate

Interestingly, we found a yearly reduction of 35.1% in ICU re-infection rate. To our knowledge, so far, six randomised controlled trials assessed the utility of PCT in tailoring antibiotic therapy in septic ICU individuals [10,13,15-17,28]. Only Nobre and Bouadma provided data of the re-infection rate [10,17]. Nobre and colleagues included only a small number of patients (n=79) of a mixed ICU population and it remains unclear how many surgical patients were included. However, no differences in terms of re-infection rate in the PCT-group compared to the control group were found. Bouadma and colleagues showed in 621 septic ICU patients that a PCT-guided algorithm can reduce the days on antibiotic therapy. Shortening the therapy did not affect the re-infection rate. However, less than 10% of the population were surgical ICU patients. Our findings probably may be associated with effects from an antimicrobial stewardship program with local guidelines for initial empirical antibiotic treatment and routinely clinical rounds with an infectious diseases (ID) fellow.

### Mortality and mechanical ventilation

Furthermore, an average yearly mortality reduction of 22.4% and decreased ventilation hours cannot be solely explained by implementation of a PCT algorithm. In 2004, sepsis guidelines for management of severe sepsis and septic shock were published and extensively implemented in clinical practice [29]. Impact of interventions from these guidelines or other implemented strategies during the study period cannot entirely be excluded in an observational study of such a long period, even if an extensive adjustment for covariates took place. However, although these international guidelines recommend a length of 7 to 10 days for antibiotic therapy in patients with severe sepsis, in two recent German multi-center studies, duration of antibiotic treatment was quite longer. In the VISEP study (2003 – 2005) [30], patients in the different study groups with a median stay of 13.5 to 16.0 days in the ICU had a median of zero antibiotic-free days. Similarly, also a median of zero antibiotic-free days was found in the MAXSEP study (2007 – 2010) [31] where patients had a median ICU-LOS of 12 days.

### Antimicrobial stewardship

PCT-algorithms for guidance of antibiotic treatment may enhance adherence to guidelines since clinicians may be reassured in their decision to discontinue antibiotics by an objective parameter. It must be emphasised that PCT-algorithms are only a single component in strategies for optimising antibiotic treatment. Antimicrobial stewardship programs are important concepts for a more rational utilisation of antibiotics [32]. Most elements of antimicrobial stewardship programs focus on structural and professional improvements: educational programs, local guidelines for an appropriate initial choice of antibiotics, resistance statistics and strategies (e.g. clinical rounds with infectious diseases fellow) for streamlining and de-escalating antimicrobial therapy. In this context, integration of biomarker-guided antibiotic treatment into antibiotic stewardship programs appears to be essential [33].

### Cost effectiveness

Our study revealed no significant antibiotic cost reduction. But there seems to be a trend to lower costs over the years. In our hospital, cost amounted to 14 Euros per PCT-test. But, a final assessment of cost effectiveness of a PCT-guided antibiotic therapy cannot be made with the present study design since there are numerous variables influencing clinical processes of care, many of which are difficult to quantify. For example, multi-resistant microorganisms significantly increase treatment expenses due to requirement of isolation procedures and use of costly reserve antibiotics. Shortening antibiotic treatment may have potential to reduce the incidence of multi-resistant microorganisms and therefore consequential costs. Also, possibly reduced incremental cost of antibiotic related adverse effects were not considered in our study. Furthermore, in times of shortage of ICU beds, a reduction in ICU-LOS leads to economical advantages. An average yearly reduction of 2.7 days in ICU-LOS like in our study has potential to conserve resources and enhance ICU capacity and thus may increase the hospital’s refund. A previous study confirmed that under certain, assumptions managing antibiotic treatment with PCT-guided algorithms may reduce overall cost of care [34] and a data modelling analysis calculated possibly savings of € 1,163 for ICU-patients in the G-DRG system by guiding antibiotic treatment with a PCT-algorithm [35]. Nonetheless, the cost effectiveness of PCT-guided strategies needs to be fully evaluated in randomised controlled trials.

### Antimicrobial resistance

Although antibiotic use has been reduced by PCT-guided strategies, a corresponding reduction in isolation of antibiotic-resistant organisms has not yet been demonstrated [33]. Data for correlation between the reduced use of antibiotics and impact on a decrease in multi-resistant organisms were not obtained in our analysis and further studies are required to answer this question.

### PCT algorithms

Numerous PCT-algorithms with different cut-off values for starting or discontinuing antibiotic therapy have been evaluated [10-12,14,16,17,28,36-39]. Most of these trials have shown the effectiveness of standardised algorithms to guide antibiotic therapy, even if the same algorithm was used for different causes of infection. In future, further studies are required to answer the question whether we need specific PCT-algorithms for numerous different causes of severe infection in surgical patients or whether it suffices to use a general PCT-algorithm to achieve a more judicious use of antibiotics.

Nonetheless, our data and the results of several studies and meta-analyses provide that PCT-guided algorithms seem to be a useful and safe tool in clinical practice to discontinue antibiotic treatment without negative effects on clinical or economical outcomes.

### Limitations

The main shortcoming of our study is that we did not compare our results with data from a historical control group. Thus, it remains unclear to which extent reductions in antibiotic consumption and improvements in outcomes are exactly related to the PCT protocol. However, data from 2005, when the PCT-algorithm was implemented and number of PCT-measurements per patient was low, were used as baseline, showing how antibiotic use improved over the years when the program was transferred into clinical practice.

In our study 3.4% of all patients were reported to have severe sepsis whereas prevalence for severe sepsis including septic shock in Germany’s ICUs is about 11% [40]. A retrospective search for patients with severe sepsis in our ICU database depends on quality of primary documentation and possibly not all eligible patients could be identified and included. This may explain the seemingly low number of patients detected with severe sepsis in our study. In addition, our ICU was also used for short-term postoperative and intermediate care and therefore the rate of patients with the highest risk for infections was probably low. Nevertheless, a higher total number of patients with severe sepsis probably would have increased the validity of our study.

## Conclusions

This is the first study that analyses the long-term effects on antibiotic consumption after implementation of a PCT-guided algorithm to discontinue antibiotic treatment under real-life clinical conditions in ICU-patients. In a real-life clinical setting, implementation of a PCT-protocol was associated with a reduced duration of antibiotic therapy in septic ICU patients without compromising clinical or economical outcomes. Further studies to assess the impact of PCT-protocols on reduction of resistant organisms are required. Also, algorithm-adherence and cost-effectiveness of a procalcitonin-guided strategy should be fully evaluated in a real-life setting of an ICU.

## Abbreviations

ACCP: American college of chest physicians; ANCOVA: Analysis of covariance; ANOVA: Analysis of variance; APACHE: Acute physiology and chronic health evaluation score; CI: Confidence interval; FiO2: Fraction of inspired oxygen; G-DRG: German diagnosis related group; ICU: Intensive care unit; LOS: Length of stay; PaO2: Partial pressure of arterial oxygen; PCT: Procalcitonin; RCT: Randomised controlled trial; SAPS: Simplified acute physiology score; SCCM: Society of critical care medicine

## Competing interests

SS served as consultant and has received payments from BRAHMS AG for speaking engagements. KW has received funding from BRAHMS AG for performing the statistical analysis. All other authors declare no competing interests.

## Authors’ contribution

AH, SS and MH carried out data collection, contributed to the design of the study and drafted the manuscript. BB participated in the design of the study. KW participated in the design of the study, performed the statistical analysis and drafted the manuscript. AG and KB participated in the design of the study and data collection. All authors read and approved the final manuscript.

## Pre-publication history

The pre-publication history for this paper can be accessed here:

http://www.biomedcentral.com/1471-2334/13/158/prepub
